# Sexual dysfunction in Assyrian/Syrian immigrants and Swedish-born persons with type 2 diabetes

**DOI:** 10.1186/1756-0500-5-522

**Published:** 2012-09-24

**Authors:** Marina Taloyan, Alexandre Wajngot, Sven-Erik Johansson, Jonas Tovi, Kristina Sundquist

**Affiliations:** 1 Center for Primary Health Care Research, Region Skåne/Lund University, CRC, hus 28, plan 11, Jan Waldenströms gata 35, SUS, 205 02 Malmö, Sweden; 2 Stress Research Institute, Stockholm University, SE −106 91 Stockholm, Sweden; 3 Karolinska Institutet, Center for Family and Community Medicine, Alfred Nobels allé 12, SE −141 83 Huddinge, Sweden

**Keywords:** Sexual dysfunction, Diabetes type 2, Immigrants, Sweden

## Abstract

**Background:**

Few studies have investigated sexual dysfunction in immigrant patients with type 2 diabetes in Sweden. The aim of this study was to examine the association between ethnicity and sexual dysfunction and to analyze if this association remains after adjusting for explanatory variables including age, marital status, HbA1c, triglycerides, and hypertension. This cross-sectional study was conducted at four primary health care centers in the Swedish town of Södertälje. A total of 354 persons with type 2 diabetes (173 Assyrians/Syrians and 181 Swedish-born patients) participated in the survey. The main outcome measure was the self-reported presence of *sexual dysfunction* based on two questions, one regarding *loss of ability to have sexual intercourse* and the other *loss of sexual desire.* Response rates were 78% and 86%, respectively.

**Findings:**

The total prevalence of loss of ability to have intercourse was 29.5%. In the multivariate models, the odds of loss of ability to have intercourse was significantly higher in the oldest age group (OR = 5.80; 95% CI, 2.33–14.40), in men (OR = 3.33; 95% CI, 1.33–8.30), and in unmarried individuals (OR = 2.40; 95% CI, 1.02–5.70). The odds of reporting loss of sexual desire was higher in Assyrians/Syrians than in Swedish-born patients and increased from 2.00 in the age- and gender-adjusted model to 2.70 in the fully adjusted model when all confounders were taken into account.

**Conclusions:**

Sexual dysfunction appears to be more common in Assyrians/Syrians than in Swedish-born patients. Health care workers should actively ask about sexual function in their patients with type 2 diabetes.

## Findings

### Background

Individuals with type 2 diabetes have a higher prevalence of sexual dysfunction than those without type 2 diabetes [1,2]. Although type 2 diabetes is more common among some immigrant groups, few studies have investigated sexual dysfunction in immigrants with type 2 diabetes. One of these few studies was a Swedish study exploring beliefs about health and illness in men with diabetes [3]. This study showed that sexual function was one of the most important health-promoting factors for Arab men and men from the former Yugoslavia, whereas it was less important among Swedish men [3]. Most studies on the topic have investigated men and women separately. For instance, many studies have focused on erectile dysfunction (ED) in men [4], which has been recognized as a complication of diabetes. One study in 1312 Korean men with type 2 diabetes concluded that they had a six times higher prevalence of ED than the general male population [5]. Malavige et al. showed that ED in men in Sri Lanka was strongly associated with reduced libido [6].

The effects of diabetes on sexual function have been less thoroughly studied in women than in men [4], although there is a growing number of studies of female sexual dysfunction. In Malaysian women, older age, longer marriage, having many children, and having a level of higher education were risk factors for sexual dysfunction [7]. Young Turkish women with type 2 diabetes (mean age: 38.8 years) had a higher prevalence of lack of libido than age-matched healthy women (77% vs. 20%, respectively) [8].

A Swedish study assessing sexual function in patients with chronic disorders found that general sexual functioning decreased with age and was most common in patients with diabetes, with loss of erection function a dominant factor [9]. This was explained by the presence of microvascular complications, psychiatric disease, and treatment with nitroglycerine. The same study found that diabetes was a secondary risk factor for decreased sexual function in patients with angina pectoris [9].

In Sweden there are approximately 70,000 to 80,000 Assyrians/Syrians [10], originating mainly from Turkey, Syria, Iraq, and Lebanon. About 20,000 Assyrians/Syrians live in the town of Södertälje. Assyrians/Syrians are an ancient ethnic group from Mesopotamia whose Christian religion is an important part of their identity [11]. Assyrians belong to one of four churches: Syrian-Orthodox, Nestorian, Chaldeian, and Syrian-Catholic. Individuals from the Syrian-orthodox group and those who do not want to be defined as Assyrians may identify themselves as Syrians [12]. Thus, both terms were used in the present study.

Few studies have investigated sexual function/dysfunction in immigrants with type 2 diabetes in general or in Assyrians/Syrians with type 2 diabetes in particular. As sexual health is an important issue and patients themselves might be embarrassed to tell their doctor about their sexual dysfunction, this study aimed to fill this gap in the literature. To the best of our knowledge, this is the second study on self-reported sexual health and the first on self-reported sexual dysfunction in a population consisting of Assyrians/Syrians and Swedes. Our first study in the same Assyrian/Syrian sample investigated dissatisfaction with one’s sexual life compared to Swedish-born patients with type 2 diabetes [13]. That study showed no significant ethnic differences in the dissatisfaction with one’s sexual life [13].

The first aim of the current study was to examine whether there is an association between ethnicity and self-reported sexual dysfunction in patients with type 2 diabetes. The second aim was to analyze whether this association remained after adjusting for explanatory variables including age, gender, marital status, raised triglyceride levels, and hypertension.

## Methods

### Study setting and participants

This study is based on the same survey that was used to investigate ethnic differences in dissatisfaction with one’s sexual life and published in BMC Public Health (2010) [13]. The participants were consecutively selected from the registers of patients with type 2 diabetes at four primary health care centers in Södertälje. A total of 354 persons were included in the survey: 173 Assyrians/Syrians and 181 Swedes. Ethnicity is discussed further in the section on explanatory variables below. Medical information and laboratory data were gathered from the patient records of all participants.

### Main outcome measure

The main outcome measure was the presence of *sexual dysfunction*. Presence of sexual dysfunction was ascertained on the basis of the participants’ answers to two questions about sexual life that were asked as part of a questionnaire on type 2 diabetes in Assyrians/Syrians and Swedish-born individuals in Södertälje. Those questions were not a part of a validated questionnaire and the purpose of including them in the health survey was to study subjective perceptions of type 2 diabetes patients about their sexual life.

The first question was “Do you have the ability to have sexual intercourse?” Responses were divided into two groups: “yes” and “no”. The second question was “Do you have any sexual desire?”, the answers to which were categorized as “yes” or “no”. Prior to asking the two questions on sexual life, we stated to the participants that these particular questions may be perceived as being sensitive, but we asked them to answer them as well as they could.

### Explanatory variables

**Age** was divided into three groups: 32–59, 60–69, and ≥ 70 years. There were similar numbers of patients in the groups.

**Self-reported ethnicity** was defined as Assyrian/Syrian or Swedish-born. The Swedish-born group was ethnically homogeneous. The Assyrian/Syrian ethnic group included both first- and second-generation immigrants; that is, individuals born abroad and individuals with at least one parent who was born abroad.

There is no registration of ethnicity in official Swedish statistics; rather, immigrants are identified by country of birth, parents’ country of birth, and/or citizenship. For this reason, the identification of potential participants by ethnicity took place as follows: First, we created a list of the patients with diabetes type 2 at each of the four participating primary health care centers. Second, we identified Swedes and Assyrians/Syrians based on the patients’ surnames and the personnel’s personal knowledge of the patients. Then, each health care center’s personnel contacted prospective participants by phone and invited them to fill out the questionnaire at the primary health care center. The participants were interviewed face-to-face at the primary health care centers after verbal agreement by phone. One of the questions in the questionnaire was about ethnicity. Two persons who identified themselves as neither Assyrian/Syrian nor Swedish-born were excluded from the study.

**Marital status** was divided into two groups: 1) married or cohabiting and 2) living alone or with children only. We combined those living alone with those living with children because of the small number of participants among Assyrians/Syrians who were living alone.

**HbA1c** was divided into two groups: normal (≤ 6.0%) and high (> 6.0%) (Swedish mono-S method [14]).

**Triglycerides** were divided into normal (< 1.7 mmol/L) and increased (≥ 1.7 mmol/L) levels.

### Hypertension

*Systolic blood pressure* was dichotomized as normal (≤ 130 mmHg) and high (> 130 mmHg). *Diastolic blood pressure* was divided into normal (≤ 80 mmHg) and high (> 80 mmHg). If systolic blood pressure was > 130 mmHg and/or the diastolic blood pressure > 80 mmHg, the patient was considered to be hypertensive; otherwise, the patient was considered to be non-hypertensive.

### Statistical analyses

Estimation of the prevalence of the outcome variable and determination of the differences in socio-demographic and medical characteristics between the two ethnic groups were performed using several tests. The Kolmogorov-Smirnov test was used to explore the parameter distribution. Correlations were assessed by using Pearson’s and Spearman’s methods for normally and non-normally distributed data. In addition, the unpaired two-sided Student’s *t* test was used to compare the means of normally distributed parameters. For comparison of non-normally distributed parameters, Mann–Whitney *U* test was performed. The statistical software used was Stata version 9 [15]. Unconditional logistic regression analysis was applied to estimate the odds ratios (ORs) and 95% confidence intervals (CIs) for the associations between sexual dysfunction and the explanatory variables. We created two models to assess differences in lack of sexual ability: one model adjusted for age and sex and the other, fully adjusted model included age, sex, marital status, HbA1c, triglycerides, and hypertension. We lacked information on menopausal status and therefore did not control for this variable. Reference groups were the following: age 32–59 years, male sex, being married/cohabiting, HbA1c < 6%, raised triglycerides, and having hypertension. The fit of the models was judged by the Hosmer-Lemeshow goodness-of-fit test [16].

### Ethical considerations

The study was approved by the regional ethical committee at Karolinska Institutet (reference number 2006/4:8, 2006-09-27). The respondents provided verbal informed consent for participation in the study and for the gathering of data from patient records. They gave the same verbal consent to participate on two occasions: once by phone and once before starting the face-to-face interviews at the primary health care centers.

## Results

In total, 354 participants filled out the questionnaire. The Swedish population was somewhat older, with a mean age of 64 years (range: 32–86 years), as shown in Table 1. The Assyrian/Syrian population was younger with a mean age of 61 years (range: 32–83 years). There seemed to be ethnic differences in the duration of type 2 diabetes, which was self-reported and based on the age of the participants when the diagnosis was made, but these differences were not statistically significant. The same pattern was observed for diabetes control with a mean HbA1c of 6.1% (SD 1.2) in Swedish-born patients and 6.3% (SD 1.6) in Assyrians/Syrians.

**Table 1:** Distribution (%, n) and anthropometric data (means; standard deviations) of background variables in Assyrian/Syrians and Swedes patients, n = 354

**Sociodemographic variables**	**Assyrians/ Syrians n = 173**	**Swedes n = 181**	**Test of difference P value**
Total	48.9	51.1	
**Gender**			
Female	48.5 (84)	44.2 (80)	0.411^a^
Male	51.5 (89)	55.8 (101)	
**Age (years)**			
32-57	39.3 (68)	27.1 (49)	**0.003**^**b**^
58-70	37.0 (64)	41.4 (75)	
> 70	23.7 (41)	31.5 (57)	
**HbA1c (MD)**	6.0	5.8	0.28^c^
**(>6%)**	49.7	40.6	0.10^c^
**Smoking habits** (yes)	16.9 (29)	18.3 (29)	0.63^a^
**Anthropometric data**			
**Body mass index (MD)**	31.3	29.1	0.0049^b^
**BMI (kg/m**^**2**^**)**			
Normal (<25)	11.8 (20)	15.0 (27)	**0.017**^**c**^
Overweight (25.0 – 29.9)	30.6 (52)	42.5 (76)	
Obese (≥ 30)	57.6 (98)	42.5 (76)	
**Duration (age of onset) (MD)**	54	56	0.096^c^
30-45	29.0 (42)	21.0 (30)	0.090^b^
46-60	60.0 (87)	61.0 (89)	
>60	11.0 (16)	18.0 (27)	

^a^ Pr-test.

^b^ Chi2-test.

^c^ Mann–Whitney tes.

Table 2 presents the total prevalences of the outcome variables; 78% of participants (n = 273) answered the first question about their ability to have sexual intercourse. Among Assyrians/Syrians the response rate was 65% (n = 112), and among Swedish-born patients the response rate was 89% (n = 161). The total prevalence of lack of ability to have intercourse was 29.5%. No significant differences were noted between the ethnic groups: the prevalence was 28.3% among the Assyrians/Syrians and 30.4% among the Swedish-born respondents. On the other hand, there were differences according to sex: 76.5% of men and 23.5% of women reported loss of ability to have sexual intercourse.

The total response rate for the question about sexual desire was 86% (n = 301). Among Assyrians/Syrians the response rate was 76% (n = 132), and among Swedish-born patients the response rate was 93% (n = 169). Significant differences were noted in loss of sexual desire only between Assyrian/Syrian and Swedish women.

In Table 3, ORs for loss of ability to have sexual intercourse are shown in a model adjusted for age and sex and in a final model. After adjusting for age and sex, the odds of loss of ability to have intercourse were 5.00 times higher in those ≥ 70 years of age (95% CI, 2.10–11.40) than in those 32–59 years old. Furthermore, men had higher odds of reporting loss of ability to have sexual intercourse than women (OR = 3.01; 95% CI, 1.30–7.23). In the fully adjusted model, the odds of reporting loss of ability to have sexual intercourse was higher in those aged ≥ 70 years (OR = 5.80; 95% CI, 2.33–14.40), in men (OR = 3.33; 95% CI, 1.33–8.30). Moreover, the results for the full model showed that those living alone or with children had higher odds of reporting loss of ability to have sexual intercourse (OR = 2.40; 95% CI, 1.02–5.70) than those who were married or cohabiting.

As shown in Table 4, the odds of reporting loss of sexual desire were two times higher in the Assyrian/Syrian group than in the Swedish-born participants in the age- and sex-adjusted model (OR = 2.00; 95% CI, 1.02–4.00). Both the older age groups (60–69 years and ≥ 70 years) had higher odds of reporting loss of sexual desire (OR = 2.20 and OR = 4.50, respectively) than the youngest age group. Women had more than seven times higher odds of loss of sexual desire than men (OR = 7.10, 95% CI, 3.70–13.60).

In the fully adjusted model, the ORs for reporting loss of sexual desire remained higher for the oldest age group (OR = 4.60; 95% CI, 2.00–11.00) and for women (OR = 6.54; 95% CI, 3.40–13.00), regardless of ethnicity. Furthermore, those who lived without a partner had higher odds of loss of sexual desire than married individuals (OR = 2.42; 95% CI, 1.20–5.01). The odds of loss of sexual desire were significantly higher in Assyrians/Syrians than in Swedish-born subjects (OR = 2.70, 95% CI, 1.30–5.50). The models were considered acceptable if p was < 0.05 in a likelihood ratio test. All models met this demand [16].

## Discussion

In this study, the self-reported loss of ability to have intercourse was not associated with ethnicity. On the other hand, self-reported loss of sexual desire was independently associated with ethnicity (Assyrian/Syrian vs. Swedish-born) in the fully adjusted model. In addition, loss of ability to have intercourse was independently related to older age, male sex, and living without a partner.

Advanced age is a risk factor for poor general health [17,18] and diabetes [19]. The sexual lives of individuals are influenced by several health and socio-demographic factors. According to studies investigating this subject, the following are risk factors for the absence of a normal sexual life in diabetic individuals: sexual dissatisfaction, lack of orgasm/erection, low sexual arousal, lack of lubrication, and sexual pain. A study of 230 married Malaysian women showed that factors such as older age, being married more than 14 years, and having less sexual intercourse were associated with a lack of lubrication [7]. A large study of 7,243 healthy middle-aged women aged 40–59 found that the prevalence of sexual dysfunction was high but differed between different populations. The most important associated risk factor was a decrease in vaginal lubrication. Additionally, and in contrast to the study of Malaysian women, higher educational level protected against sexual dysfunction [20]. The female participants of Assyrian/Syrian origin in the present study have a low educational level and 20% of the Assyrian/Syrian participants are illiterate [25% of women and 14.6% of men], which may highlight the need for prospective studies exploring the association between educational level and sexual life.

Sexual dysfunction in women might be related to the menopausal status and the negative impact of menopause on sexuality [21,22]. A study in American women aged 30 to 70 years who had been in stable relationships for more than 3 months concluded that the prevalence of low sexual desire is higher in menopausal women than in premenopausal women [23]. Another study assessing the prevalence of sexual dysfunction in premenopausal women showed that, compared to the control group, those with the metabolic syndrome had reduced sexual function [24]. The associations between sexuality, hyperglycemia, elevated body weight, and the metabolic syndrome are also strong in menopausal women [25].

Relationships between hyperglycemia and higher BMI and lipid abnormalities were observed in several studies [26-28]. Despite the fact that 48% of the participants in the current study had BMI values higher than 30 kg/m^2^, the BMI variable was not a statistically significant confounder. Our results are based on self-reporting, and physical health and culture may have influenced the responses to sensitive questions on sexuality. It is important to note that, due to cultural and religious values, individuals with an Assyrian/Syrian ethnic background were presumed to only have a sexual life within the context of marriage [12].

Assyrians/Syrians are an ancient ethnic group from Mesopotamia. The Christian religion is one of the important parts of their identity [29]. The majority of the participants in the present study accepted their diabetes as being sent by God and the separation from or loss of a life partner was not followed by another partner. “We do not do that”, said the separated woman when we asked whether she had a sexual partner or not. Perhaps this was one of the reasons for the proportion of Assyrians/Syrians who responded to this question being smaller compared to the proportion of Swedish-born participants. On the other hand, patients’ perceptions of sex and sexual practices may be individual. This might be investigated more deeply using valid and standardized scales on sexual life and sexual function.

### Strengths and limitations

This study has several strengths. Registration by ethnicity does not occur in the official Swedish statistics, where immigrants are identified according to country of birth, parents’ country of birth, and/or citizenship. A unique feature of this study is the use of data on self-reported ethnicity and its main strength is that it is the first study of sexual life in Assyrians/Syrians with type 2 diabetes. Another strength of this study is the inclusion of patients from several health care centers; the sample can therefore be considered highly representative of Assyrians/Syrians in Södertälje.

One limitation of the study is that the instrument used to explore sexual dysfunction has not been validated. Another limitation is that it assessed Assyrian/Syrian patients with type 2 diabetes living in one town and the results cannot therefore be generalized to the entire Assyrian/Syrian population with diabetes in Sweden. A third limitation is that the cross-sectional nature of the study and the relatively small sample size precluded the possibility of drawing extensive causal conclusions.

## Conclusions

Loss of sexual desire was independently associated with ethnicity (Assyrian/Syrian vs. Swedish-born) in the fully adjusted model. Sexual function is an important part of general health status and quality of life and should be included in the clinical evaluation of patients with type 2 diabetes. The results of this study suggest that physicians and other health care workers should feel encouraged to ask about sexual function in their patients, regardless of the patients’ ethnic background, age, or sex.

## Abbreviations

CI: Confidence interval; GP: General practitioner; OR: Odds ratio.

## Competing interests

The authors declare that they have no competing interests.

## Authors’ contributions

MT conceived the idea for the survey. MT, AW, JT, SEJ and KS designed the study. MT and SEJ performed the statistical analysis. MT drafted the manuscript. KS, AW, JT and SEJ revised the manuscript. All authors read and approved the final manuscript.
